# Evolutionary mechanisms of habitat invasions, using the copepod *Eurytemora affinis* as a model system

**DOI:** 10.1111/eva.12334

**Published:** 2015-11-30

**Authors:** Carol Eunmi Lee

**Affiliations:** ^1^ Center of Rapid Evolution (CORE)University of WisconsinMadisonWIUSA

**Keywords:** antagonistic pleiotropy, balancing selection, biological invasions, crustacea, evolutionary trade‐offs, immunohistochemistry, osmoregulation, phenotypic plasticity

## Abstract

The study of the copepod *Eurytemora affinis* has provided unprecedented insights into mechanisms of invasive success. In this invited review, I summarize a subset of work from my laboratory to highlight key insights gained from studying *E. affinis* as a model system. Invasive species with brackish origins are overrepresented in freshwater habitats. The copepod *E. affinis* is an example of such a brackish invader, and has invaded freshwater habitats multiple times independently in recent years. These invasions were accompanied by the evolution of physiological tolerance and plasticity, increased body fluid regulation, and evolutionary shifts in ion transporter (V‐type H^+^ ATPase, Na^+^, K^+^‐ATPase) activity and expression. These evolutionary changes occurred in parallel across independent invasions in nature and in laboratory selection experiments. Selection appears to act on standing genetic variation during invasions, and maintenance of this variation is likely facilitated through ‘beneficial reversal of dominance’ in salinity tolerance across habitats. Expression of critical ion transporters is localized in newly discovered Crusalis leg organs. Increased freshwater tolerance is accompanied by costs to development time and greater requirements for food. High‐food concentration increases low‐salinity tolerance, allowing saline populations to invade freshwater habitats. Mechanisms observed here likely have relevance for other taxa undergoing fundamental niche expansions.

## Introduction: major habitat transitions

Colonizations from marine to freshwater habitats, followed by terrestrial colonizations, represent among the most dramatic evolutionary transitions in the history of life (Hutchinson 1957; Little 1983, 1990; Smith and Szathmáry 1998; Miller and Labandeira 2002). As life evolved in the sea, colonizations into freshwater and terrestrial habitats impose profound physiological challenges for most taxa (Hutchinson 1957; Lee and Bell 1999). Such transitions have been relatively rare, such that the vast majority of animal diversity still remains in the sea. For instance, of the ~35 animal phyla, only 16 have invaded fresh water and only 7 have been able to invade land (Little 1983, 1990).

Marine animals, aside from vertebrates, tend to possess body fluids that resemble the surrounding seawater in ionic composition. Thus, colonizing dilute environments imposes great challenges for acquiring essential ions against steep concentration gradients (Beyenbach 2001; Morris 2001; Tsai and Lin 2007; Lee et al. 2011). Overcoming these challenges was critical for freshwater colonizations, which then provided key adaptations enabling the colonization of land (Wolcott 1992; Anger 2001; Morris 2001; Glenner et al. 2006). Challenges imposed by these habitat transitions have led to the evolution of a variety of key innovations in different taxa. In general, transitions into more stressful environments have been accompanied by the evolution of increased physiological regulation and homoeostasis. In the case of colonizations departing from the ancestral sea, evolution of increased ionic and osmotic regulation was required to maintain homoeostasis, to approach an internal ocean against the nonmarine environments (Withers 1992; Willmer et al. 2004; Charmantier et al. 2009).

While such major habitat transitions have been, and continue to be, relatively rare, some invasive species are extraordinary in their ability to breach such habitat boundaries (Lee and Bell 1999; Lee and Gelembiuk 2008; Lee 2010). In recent years, many species have invaded fresh water from saline (i.e. marine or brackish) habitats as a result of human activity (Lee and Bell 1999; Ricciardi and MacIsaac 2000). Invasions from saline waters into freshwater lakes and reservoirs have been facilitated by the construction of canals, the advent of boat traffic, and ship ballast and bilge water discharge into freshwater lakes. In addition, there have been frequent instances of inadvertent freshwater invasions through the stocking of lakes and reservoirs with anadromous fish (such as striped bass), and deliberate large‐scale introductions of brackishwater invertebrates to support freshwater sport fisheries (US Fish and Wildlife Service 1965; Jażdżewski 1980; Bij de Vaate et al. 2002).

A remarkable feature of these invaders is that many of these originally saline populations have become among the most successful and explosive invaders in freshwater habitats. For example, the vast majority of recent invaders into the North American Great Lakes have originated from more saline waters, such as from the adjacent coast, as well as from more distant brackish bays and seas (Lee and Bell 1999; Ricciardi and MacIsaac 2000). These invaders include many brackishwater species from the Black and Caspian Sea region, such as the zebra mussel *Dreissena polymorpha*, the fishhook water flea *Cercopagis pengoi* and the hydroid *Cordylophora* spp., as well as a large number of amphipods (Witt et al. 1997; Cristescu et al. 2001, 2003, 2004; Gelembiuk et al. 2006; May et al. 2006; Folino‐Rorem et al. 2009). In fact, many of the species interactions native to the Black and Caspian Sea ecosystem are becoming reconstituted in the North American Great Lakes (Ricciardi and MacIsaac 2000; MacIsaac et al. 2001).

Such invasions are remarkable, given that saline and freshwater invertebrates are typically separated by a biogeographic boundary, across which most species are physiologically unable to penetrate (Khlebovich and Abramova 2000). Typically, intermediate salinities of ~5 PSU (≈150 mOsm/kg) separate saline and freshwater species. Yet, explosive freshwater invasions by brackishwater species are taking place despite the fact that these species are inefficient osmoregulators in freshwater habitats (Taylor 1985; Taylor and Harris 1986; Horohov et al. 1992; Dietz et al. 1996; Lee and Petersen 2003; Lee et al. 2003). How are these invaders able to survive these radical habitat shifts? Might common mechanisms govern the ability to invade fresh water from more saline sources? Why do many invaders originate from particular geographic locations, such as the Black and Caspian Sea region?

Early observations of contemporary saline to freshwater invasions and rapid evolution during these invasions (Lee 1999; Lee and Bell 1999) were surprising and countered commonly held views of the time. Invasive species were often regarded as immutable entities that were able to traverse habitat boundaries through their broad tolerance or phenotypic plasticity alone, without the need to invoke evolution (Wolff 2000). Alternatively, some argued that freshwater invasions by Ponto‐Caspian and other brackish species occurred a long time ago (e.g. before 1800), rather than as recent human‐mediated events, based on distribution records and erroneous taxonomic designations (see section below on, *Invasive species as sibling species complexes and cryptic invasions*) (Strayer 1999; Wolff 2000). Such perspectives have since been countered by numerous detailed studies that have documented geographic pathways of recent invasions, elucidated systematics of cryptic invasive species complexes and revealed rapid physiological evolution during contemporary invasions for a wide variety of taxa (Cristescu et al. 2001, 2004; Väinölä and Oulasvirta 2001; Bij de Vaate et al. 2002; Hebert and Cristescu 2002; Lee 2002, 2010; Lee et al. 2003, 2007, 2011, 2012, 2013; Gelembiuk et al. 2006; May et al. 2006; Lee and Gelembiuk 2008; Winkler et al. 2008; Folino‐Rorem et al. 2009; Leal and Gunderson 2012; Urbanski et al. 2012; Kolbe et al. 2014; Rewicz et al. 2015).

As invasive populations represent the rare victors during habitat shifts, they provide valuable models for understanding mechanisms of niche evolution and responses to diverse forms of environmental change, such as global climate change. In addition, invasive populations are particularly useful for studying physiological evolution because the process of evolutionary change could be observed directly, in real time. Furthermore, invasive populations are valuable as models for studying the incipient stages of adaptation in response to environmental change (Lee 2010).

The term ‘invasive species’ has several usages, but typically refers to nonindigenous species that have adverse impacts in the habitats they invade, in terms of threats to ecology, economy or public health. However, the impacts of *E. affinis* introductions vary considerably among populations colonizing different locations, from introductions into empty niches in newly constructed reservoirs to invading estuaries where they could displace local copepod populations and damage fisheries. Thus, when referring to *E. affinis* as ‘invasive’, this review uses a looser definition of the term, of nonindigenous populations that have become widespread in their novel habitats (with the potential to have adverse effects in some locations). The exact definition of the term is secondary to the goals of this review, as the following studies are important for analyzing evolutionary mechanisms that allow populations to colonize novel niches, regardless of their immediate ecological or environmental impacts.

This review describes research on evolutionary mechanisms of habitat invasions, using the copepod *Eurytemora affinis* as a model system, focusing on results from the Lee Lab and collaborators. This summary first discusses phylogeographic pathways of invasions, and cryptic speciation within the invasive species complex (sections *The copepod Eurytemora affinis as a model system: phylogeographic pathways of invasions* and *Invasive species as sibling species complexes and cryptic invasions*). The focus then centres on patterns and mechanisms of physiological evolution during invasions (sections *Evolution of physiological tolerance and plasticity during invasions* *,* *Fluctuating environments and the maintenance of genetic variation* *,* *Physiological evolution: evolution of body fluid regulation and the evolution of ion transporter expression and activity* and *‘It's all in the legs’: localization of ion transporter expression in the legs*). The copepod *E. affinis* exemplifies a remarkable case of rapid osmoregulatory evolution during transitions from saline to freshwater habitats. This rapid evolution is likely facilitated by the maintenance of genetic variation in the native range, providing genetic substrate upon which selection could act during invasions. Thus, *E. affinis* was used to test for the presence of ‘beneficial reversal of dominance’, which could serve as an important mechanism for maintaining genetic variation in salinity tolerance within populations (section *Fluctuating environments and the maintenance of genetic variation*). The most striking evolutionary changes during saline to freshwater invasions were the shifts in ion transporter expression and activity (section *Physiological evolution: evolution of body fluid regulation and the evolution of ion transporter expression and activity*). Localization of a key primary ion transporter involved in ion uptake was concentrated primarily in the legs, in the newly discovered Crusalis organs (section *‘It's all in the legs’: localization of ion transporter expression in the legs*). Finally, this study concludes with discussion of some costs associated with adaptation to fresh water, including life history and energetic costs (sections *Cost of adaptation? Life history evolution* and *Cost of adaptation? Energetic cost and the evolution of salinity tolerance* × *food requirement*). The strength of these studies was in analyzing evolutionary mechanisms underlying habitat transitions across multiple hierarchical levels of biological organization, from molecular, biochemical, cellular and tissue, to organismal and population levels.

## The copepod *Eurytemora affinis* as a model system: phylogeographic pathways of invasions

Copepods are common as invaders, both between and within continents (Lavoie et al. 1999; Cordell et al. 2008; Ruiz et al. 2011). Copepods arguably form the largest biomass of animals on the planet (Hardy 1970; Humes 1994; Verity and Smetacek 1996) and tend to dominate the plankton in coastal ecosystems. Thus, it is not surprising that copepods often comprise the most abundant and diverse members within ballast water assemblages (Chu et al. 1997). Copepods constitute a considerable portion of crustacean invaders into North American coastal waters (~13%), mostly through commercial shipping and the dumping of ship ballast water (Ruiz et al. 2011). Copepod invasions could potentially have large impacts on fisheries by displacing the native zooplankton prey of native fishes (Ruiz et al. 2011).

**Figure 1:**
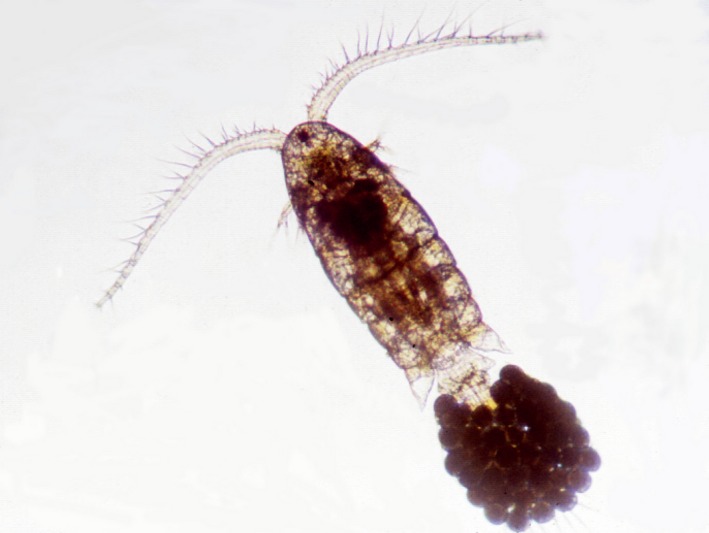
The copepod *Eurytemora affinis*. An adult female, 1.5 mm in length, is shown with a large egg sac. Photograph by Carol Eunmi Lee.

**Figure 2:**
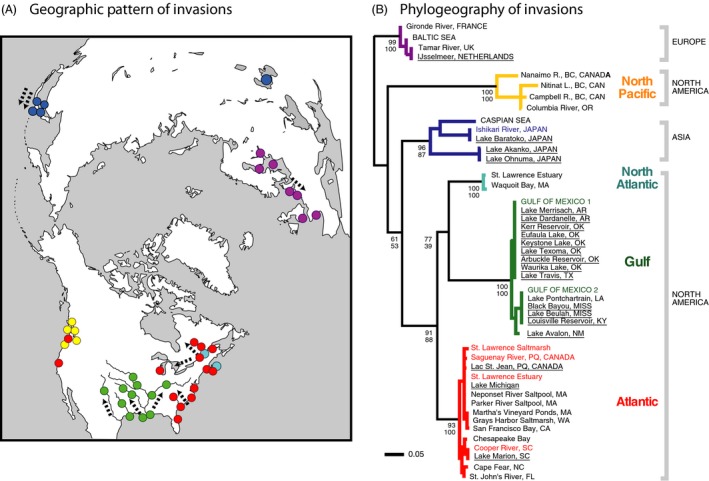
(A) Populations of *Eurytemora affinis*, showing independent invasions from saline to freshwater habitats. Colours of dots represent genetically distinct clades shown in the phylogeny in Fig. 2B. Dotted arrows represent pathways of invasions, which occurred within the past ~70 years (Lee 1999). (B) Phylogeny of *E. affinis* populations based on cytochrome oxidase I (COI, 652 bp) sequences. Locations are shown at the branch tips, with colours representing the genetically distinct clades shown in Fig. 2A. Recently derived freshwater populations are underlined. Numbers next to nodes are bootstrap values based on 1000 bootstrap replicates using distance (upper numbers) and parsimony (lower numbers) approaches. Branch lengths are maximum‐likelihood distances that account for multiple substitutions per site. Figures adapted from Lee (1999, 2000).

**Figure 3:**
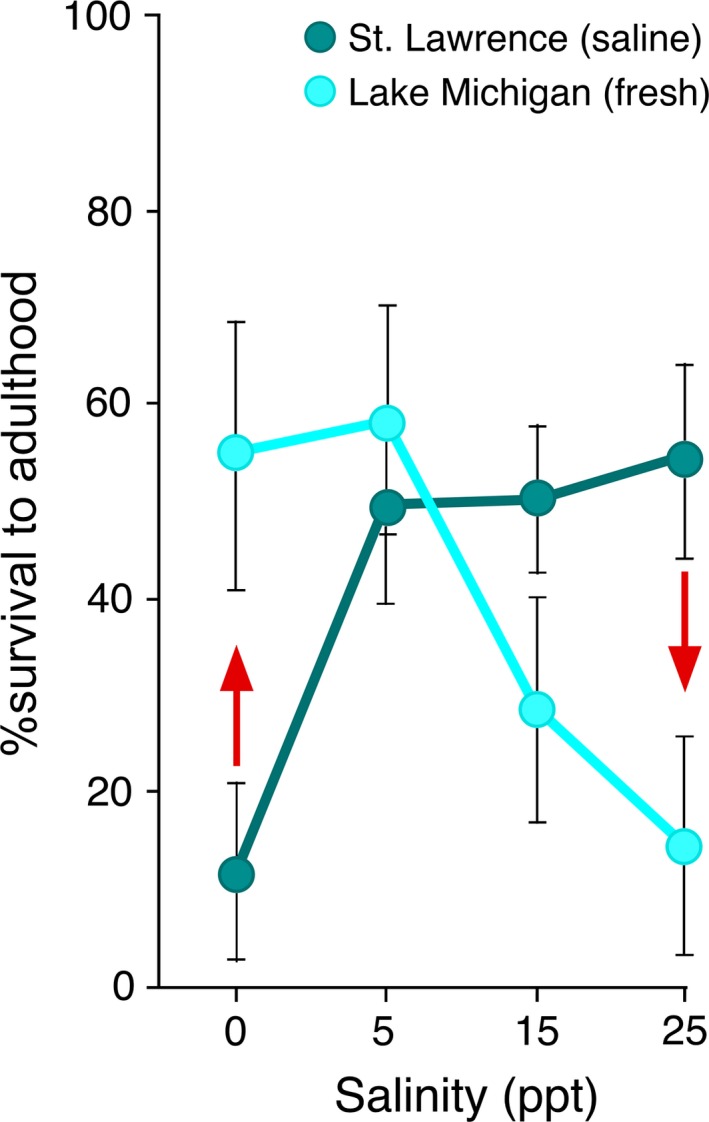
Evolutionary shifts in salinity tolerance following an invasion from saline (St. Lawrence) into freshwater (Lake Michigan) habitats (Lee et al. 2003, 2007) by the copepod *E. affinis*. Red arrows show shifts in salinity tolerance between the populations. Data are mean %survival to adulthood ± SE for 8 clutches. Figure adapted from Lee et al. (2003).

Much insight into mechanisms of invasive success has been gained through the study of the common coastal copepod *Eurytemora affinis* (Fig. 1). The study of this copepod represents among the first use of phylogeographic analysis to reconstruct geographic pathways of contemporary invasions (Fig. 2) (Lee 1999), along with a few other studies (Geller et al. 1997; Davies et al. 1999). Phylogeographic analysis of *E. affinis* populations yielded the first genetically based documentation of contemporary shifts from saline into freshwater habitats (Fig. 2B) (Lee 1999). Moreover, experimental analyses of *E. affinis* populations from their native and invaded ranges were among the first to document physiological evolution during contemporary habitat invasions (Fig. 3) (Lee 1999; Lee and Petersen 2002, 2003; Lee et al. 2003, 2007, 2011, 2012, 2013). Additionally, research on *E. affinis* has emphasized the importance of evolution in the native range as critical for determining which populations have the potential to become invasive (see section *Evolution of physiological tolerance and plasticity during invasions*) (Lee and Gelembiuk 2008; Winkler et al. 2008; Hufbauer et al. 2012; Lee et al. 2013; Posavi et al. 2014).

The native distribution of the copepod *E. affinis* spans coastal waters in the Northern Hemisphere, including estuaries and salt marshes, in North America, Europe and in Asia. This copepod is a dominant member among estuarine zooplankton, forming enormous biomasses in estuaries of up to ~2 × 10^6^ individuals/m^3^ and estimated census sizes in the billions (Heinle and Flemer 1975; Simenstad and Cordell 1985; Gulati and Doornekamp 1991; Morgan et al. 1997; Peitsch et al. 2000; Winkler et al. 2005). This copepod is a dominant grazer of algae and major food source for some of the world's most important fisheries, such as herring, anchovy, salmon and flounder (Shaheen et al. 2001; Viitasalo et al. 2001; Winkler et al. 2003; Kimmel et al. 2006).

Within the past ~70 years, this estuarine and salt marsh species has invaded many freshwater habitats throughout the world, primarily into freshwater reservoirs and lakes (Lee 1999). Phylogeographic analysis of *E. affinis* revealed that there had been at least eight independent invasions from saline into freshwater habitats on three continents, in North America, Europe and in Asia (Lee 1999). These invasions into freshwater bodies likely occurred predominantly through ballast or bilge water transport, within the waterways connecting the saline and freshwater habitats, and in some cases through the transport of fish, such as striped bass, from downstream estuaries (Anderson and Clayton 1959; Lee 1999). Introductions of *E. affinis* into the Great Lakes originated from local estuaries (likely *via* ship ballast water), rather than from the brackish waters of the Black and Caspian Seas, which were the source of many other Great Lakes invaders. These copepods are weak swimmers and cannot move upstream into fresh water without human intervention. From a physiological perspective, the rapid saline to freshwater invasions by this species was surprising, given that copepods were thought to be limited in osmoregulatory capacity, and unable to make rapid transitions from brackish (5–15 PSU) to completely freshwater habitats.

These invasions by *E. affinis,* from saline estuaries into freshwater lakes and reservoirs, arose multiple times independently from at least four genetically distinct clades (clade = monophyletic group of related taxa, in this case populations; Fig. 2, Atlantic, Gulf, Asia, Europe clades), as well as multiple times within those clades (Lee 1999). Multiple independent invasions occurred within two North American clades (Atlantic, Gulf). These invasions originated principally from brackish (5–15 PSU) estuaries into upstream freshwater reservoirs and lakes, with invasions occurring along waterways between reservoirs or lakes within drainages and no evidence of invasions between drainages.

An important point to emphasize is that populations within invasive species often vary in their ability to invade (see next section). For example, two clades of *E*. *affinis* (Fig. 2, North Atlantic, North Pacific) have apparently not given rise to any freshwater invading populations (Lee 1999, 2000; Winkler et al. 2008). This variation in ability to invade could occur even when the invasive and noninvasive clades overlap in geographic distribution. For instance, two genetically distinct clades (Fig. 2, Atlantic, North Atlantic) overlap in geographic distribution on the Atlantic coast of North America (Fig. 2A), but populations from only one of these clades (Fig. 2, Atlantic [red clade]) have been able to extend their ranges into freshwater habitats (see next section) (Lee 1999; Winkler et al. 2008).

The multiple independent invasions from saline into freshwater habitats, arising from genetically distinct but related clades, make this system ideal for exploring mechanisms of rapid evolution during invasions into novel habitats. The multiple invasions make it possible to determine whether the same evolutionary pathways are involved during independent invasions. Exploring the degree of parallelism across these independent invasions could reveal the degree to which mechanisms of adaptation are labile or constrained (Gould 1989; Bell and Foster 1994; Cooper et al. 2003). Also, comparisons between populations from invasive and noninvasive clades could reveal properties that are exclusive of invasive populations, leading to their invasive success.

The copepod *E. affinis* provides a valuable genetic model for dissecting evolutionary mechanisms of invasive success. The genomic resources available for this system make it possible to discover the genetic loci underlying trait evolution during habitat invasions. The availability of a comprehensive genome sequence (https://www.hgsc.bcm.edu/arthropods/eurytemora-affinis-genome-project) and transcriptome sequences, the small genome size (1C = ~300 Mb) (Rasch et al. 2004), short generation time (20 day at 12°C) and the relative ease of generating inbred lines (through full‐sib matings) greatly facilitate the process of associating phenotype with genotype and also of addressing a variety of core evolutionary questions.

## Invasive species as sibling species complexes and cryptic invasions

Invasive species are not monolithic and immutable entities, but tend to harbour substantial genetic and physiological diversity. Many invasive species are composed of genetically divergent populations, in some cases cryptic sibling species, as well as physiologically diverse ecotypes. The important implication of this fact is that a small sampling of populations is unlikely to be representative of all populations within an invasive species complex. And it is often the case that not all populations within an invasive species possess the capacity to invade. Additionally, these populations could differ considerably in their ability to evolve in response to environmental change (Lee and Gelembiuk 2008). These principles were well‐illustrated using *Eurytemora affinis* as a model system, and have later been found to apply to many other invasive species (see below).

The genetically distinct clades of *E. affinis* form a sibling species complex, with evidence of cryptic speciation arising both among and within the clades (Lee 2000; Lee and Frost 2002). These clades exhibit idiosyncratic patterns of speciation, showing discordant rates of molecular evolution, morphological evolution and reproductive isolation among the clades (Lee 2000; Lee and Frost 2002). All clades show roughly equivalent levels of genetic divergence, except for the North Pacific clade (Fig. 2B, yellow, in North America), which shows the greatest genetic divergence from the other clades (Lee 2000; Lee and Frost 2002). The genetically divergent clades exhibit morphological stasis, with morphologically indistinguishable secondary sex characters among clades (e.g. female genital segment, male fifth legs, male antennule), except for a small degree of morphological divergence between the European clade (Fig. 2B, purple) and other clades (Lee and Frost 2002). Pairwise intermating between populations from different clades (Fig. 2B) revealed some reproductive isolation between all the clades, in terms of F1 or F2 hybrid sterility or inviability (Lee 2000; Lee, unpublished data). Reciprocal crosses between populations from distinct clades resulted in asymmetric reproductive isolation (Lee 2000), potentially arising from asymmetric mitochondrial‐nuclear incompatibility (Willett 2011).

In addition, within their native ranges, *E. affinis* populations show considerable heterogeneity, in terms of substantial population genetic structure and physiological diversity (Winkler et al. 2008; Lee et al. 2013). Within some locations, physiologically and genetically distinct populations occur within close geographic proximity. For instance, populations from the Atlantic and North Atlantic clades of *E. affinis* both occur within the St. Lawrence estuary (Fig. 2A), but differ in salinity tolerance and starvation resistance (see section *Cost of adaptation? Energetic cost* and *the evolution of salinity tolerance* × *food requirement*) (Lee et al. 2013). Such physiological differences between the clades likely account for differences in their tendency to give rise to invading populations. This heterogeneity in the native range likely arises from microhabitat structure, with spatial heterogeneity and differences in the degree of temporal fluctuations among microhabitats (see section *Fluctuating environments and the maintenance of genetic variation*) (Lee and Gelembiuk 2008; Winkler et al. 2008).

Mounting phylogeographic and population genetic data are indicating that cryptic speciation and population heterogeneity, such as those found within *E. affinis*, might in fact be the norm rather than the exception for invasive species (Bucciarelli et al. 2002; Gelembiuk et al. 2006; Andreakis et al. 2007; Folino‐Rorem et al. 2009; Zhan et al. 2010; Bastos et al. 2011; Teske et al. 2011; Wang et al. 2011; Pérez et al. 2012; Dickey et al. 2013; Pérez‐Portela et al. 2013; Silva‐Brandão et al. 2013). For instance, many of the ‘world's worst invaders’ belong to sibling and/or cryptic species complexes, such as the whitefly *Bemisia tabaci,* the green crab *Carcinus maenus* and the rodent *Rattus rattus,* to name a few (Geller et al. 1997; Bastos et al. 2011; Dickey et al. 2013). This preponderance of cryptic and sibling species might be linked to the fact that many invasive populations arise from native habitats marked by spatial and temporal heterogeneity, resulting in an evolutionary history characterized by recent speciation events (Lee and Gelembiuk 2008). In addition, invasive species would tend to colonize broad geographic and habitat ranges, allowing the populations to undergo local adaptation and physiological diversification, at times resulting in cryptic speciation.

Cryptic speciation and population heterogeneity within invasive species have important implications for monitoring current distributions and predicting future ranges. Typically, distribution records of invasive species do not distinguish among different sibling species and ecotypes, leading to erroneous conclusions regarding current distributions of native and invading populations of interest (Strayer 1999; Wolff 2000). Furthermore, ecological niche modelling efforts that rely on distribution data from native ranges to predict potential future ranges of invasive species could be greatly compromised by failing to account for genetic and physiological heterogeneity among populations and sibling species. Different populations (or sibling species) within the native range would tend to vary in their physiological properties and in their potential to become invasive. Moreover, such modelling efforts tend not to account for the fact that many populations could evolve and expand their ranges beyond existing boundaries.

## Evolution of physiological tolerance and plasticity during invasions

The discovery of rapid physiological evolution during habitat invasions in the copepod *Eurytemora affinis* contributed to countering common views that invasive species are cosmopolitan generalists. The potential importance of evolutionary mechanisms underlying invasive success was first raised by C. H. Waddington, who organized a symposium in 1964 that resulted in the classic volume *The Genetics of Colonizing Species* (Baker and Stebbins 1965; Waddington 1965). Subsequently, questions raised by Waddington, such as the roles of genetic architecture and evolution of plasticity (and genetic assimilation) in influencing invasive success, were largely ignored for more than 30 years (with some notable exceptions; e.g. Barrett and Richardson 1986). Concrete studies that examined evolutionary mechanisms of invasive success and evolutionary potential of invading populations only ceased to become rare after ca. 2000 (reviewed in Lee 2002, 2010).

While having ‘generalist’ strategies, such as broad tolerance or phenotypic plasticity, could play important roles in range expansions during invasions (Richards et al. 2006; Davidson et al. 2011; Knop and Reusser 2012), such attributes are often insufficient in cases where habitats extend beyond the normal tolerance ranges of invading populations. For example, detailed common‐garden experiments using *E. affinis* populations found that phenotypic plasticity at the individual level alone was not sufficient to account for the ability of saline populations to invade freshwater environments (Lee and Petersen 2003; Lee et al. 2003, 2007). While *E. affinis* has among the broadest salinity ranges among invertebrates, individual *E. affinis* populations are unable to survive the entire salinity range spanning saline to freshwater habitats. An evolutionary shift at the population level is required for the physiological shift to occur, enabling the once saline populations to persist in freshwater habitats.

Increasing numbers of studies are revealing the importance of evolution of critical traits for fundamental niche expansions to occur (reviewed in Lee 2002; Lee and Gelembiuk 2008; Lee 2010). Identifying the specific traits that evolve during invasions could provide valuable information on which factors act as constraints on those invasions. Comparisons between saline and freshwater populations of *E. affinis* uncovered striking evolutionary shifts in physiological tolerance and performance associated with freshwater invasions (Lee et al. 2003, 2007, 2011, 2012, 2013). Specifically, evolution of ion transport mechanisms appears to underlie the evolution of salinity tolerance (see sections *Physiological evolution: evolution of body fluid regulation and the evolution of ion transporter expression and activity* and *‘* *It's all in the legs’: localization of ion transporter expression in the legs*).

With respect to evolution of salinity tolerance, the freshwater populations showed enhanced low‐salinity tolerance, along with reduced high‐salinity tolerance, relative to their saline progenitors (Fig. 3) (Lee et al. 2003, 2007). This pattern was also evident in the crucial ion transport enzyme V‐type H^+^ ATPase, where freshwater‐adapted populations showed increases in enzyme activity and expression in fresh water, but declines at higher salinity (see section *Physiological evolution: evolution of body fluid regulation and the evolution of ion transporter expression and activity*) (Lee et al. 2011). These patterns of gains and losses in functions during saline to freshwater invasions revealed the presence of evolutionary trade‐offs between freshwater and saline adaptation.

Such evolutionary trade‐offs were supported by the negative genetic correlations observed between salt and freshwater tolerance in both saline and freshwater populations (Lee et al. 2003, 2007). These negative genetic correlations arise from the fact that genotypes (full‐sib clutches) that have high survival under freshwater conditions do poorly under saline conditions and *visa versa*. Such a pattern suggests that the trade‐offs between freshwater *versus* saltwater tolerance result from antagonistic pleiotropy. Antagonistic pleiotropy refers to the case when a gene controls for more than one trait, where at least one of these traits is beneficial to organismal fitness and at least one is detrimental. The implication is that selection favouring freshwater tolerance would select against high‐salinity tolerance and *vice versa*. Such antagonistic pleiotropy might be critically important for facilitating the maintenance of standing genetic variation under fluctuating (i.e. temporally varying) environmental conditions (see next section) (Curtsinger et al. 1994; Posavi et al. 2014).

Evolution of physiological tolerance and performance in *E. affinis* appears to occur through natural selection acting on standing genetic variation in the native range, leading to a mean shift in performance and tolerance at the population level (Lee et al. 2003; Lee and Gelembiuk 2008). Both saline and freshwater populations of *E. affinis* possess substantial genetic variation in salinity tolerance, as well as genetic variation in plasticity (genotype by environment interaction, G × E) (Lee and Petersen 2002; Lee et al. 2003, 2007). However, levels of genetic variation are higher in the native saline range than in the freshwater population, with a greater effect of clutch (genotype) on survival and highly significant G x E in the saline population, but only marginally significant G x E in the freshwater population (Lee et al. 2003). This shift reflects some degree of canalization towards low‐salinity survival in the freshwater population.

In particular, selection on physiological tolerance appears to act on the larval (naupliar) stages, rather than on adults (Lee et al. 2003, 2007). While the adult stages could survive relatively broad salinity ranges, the larval stages of both saline and freshwater populations suffer high mortalities in response to maladaptive salinities (Lee et al. 2003, 2007). For instance, close to 100% of mortality due to freshwater conditions in the saline population occurs prior to metamorphosis (Lee et al. 2003, 2007).

The evolution of increased freshwater tolerance in larvae appears to lead to evolutionary trade‐offs in freshwater survival *versus* development rate, as increased larval tolerance of fresh water is accompanied by the reduction in larval development rate (see section *Cost of adaptation? Life history evolution*). Also, the energetic costs of osmoregulation in fresh water lead to the evolution of increased requirement for food (see section *Cost of adaptation? Energetic cost and the evolution of salinity tolerance* × *food requirement*).

If selection favouring freshwater tolerance and performance would select against high‐salinity adaptation, then why do we witness the maintenance of genetic variance for salinity tolerance and for plasticity (G x E) in the invaded freshwater range? Given the evolutionary trade‐offs between low and high‐salinity tolerance, one would expect rapid erosion of high‐salinity tolerance in freshwater habitats, yet substantial variation remains (Lee et al. 2003, 2007). Additionally, if selection is acting on standing genetic variation in the native saline range, what mechanisms are operating to maintain that variation? Evidently, some mechanism must exist to maintain genetic variation for relevant traits in both the saline and the freshwater populations (see next section).

While phenotypic plasticity alone cannot account for the shifts in physiological tolerance and performance during habitat invasions by *E. affinis* (Lee and Petersen 2003), the evolution of plasticity might be critically important for invasive success in a manner proposed by C. H. Waddington (1953) and theoretically modelled by Lande (2009). In the presence of genetic variation in plasticity (G × E), selection would favour those extreme plastic genotypes that would enable survival in the novel invaded range. This selection for the extreme genotypes would result in the transient evolution of increased plasticity, evident from an increase in reaction norm slope (Lande 2009; Chevin et al. 2010). Over an extended period of time, selection would continue to favour the extreme novel phenotype in the invaded range, but not the original phenotype in the native range, leading to the evolution of canalization for the extreme novel phenotype and the loss of plasticity over time (Waddington 1953; Lande 2009; Chevin et al. 2010).

In the case of *E. affinis*, an evolution of increased slope is evident from the pattern of ion transporter V‐type H^+^ ATPase evolution, where the freshwater populations exhibit marked evolution of increased plasticity (see section *Physiological evolution: evolution of body fluid regulation and the evolution of ion transporter expression and activity*, Fig. 7) (Lee et al. 2011). Natural and laboratory‐selected freshwater populations both exhibited the evolution of much greater slope in V‐type H^+^ ATPase activity in response to salinity, relative to the ancestral saline populations (Fig. 7). The persistence of high levels of plasticity in the freshwater populations might be due to insufficient time for canalization to have occurred. Whether or not elevated plasticity for a critical trait is observed in the invading population depends on values of certain key variables, including time since colonization (Lande 2015). The presence of genetic variation in plasticity (G × E) in *E. affinis* populations in their native range and selection on the extreme plastic genotypes during invasions appear to play an important role in enabling the populations to survive extreme conditions in the novel range (Lee and Petersen 2002; Lande 2009; Chevin et al. 2010; Lee et al. 2011). This form of adaptation *via* selection on plasticity might be a common mechanism permitting habitat shifts into radically novel environments.

## Fluctuating environments and the maintenance of genetic variation

As mentioned above, evolution of freshwater tolerance and performance appears to have occurred through selection on standing genetic variation in the ancestral saline population. Yet, given the negative genetic correlations and apparent trade‐offs between saline and freshwater tolerance, one would expect natural selection to erode genetic variation for salinity tolerance in both saline and freshwater populations. In particular, it might seem odd that genetic variation for salinity tolerance could persist in the freshwater invading populations after ~60 years (ca. 300 generations) of residence in freshwater habitats. So, how might this genetic variance for salinity tolerance be maintained?

The maintenance of genetic variation and the evolutionary potential of invasive populations are likely to be profoundly impacted by the selection regime in their native range. An interesting pattern is that invasive populations appear to arise commonly from habitats marked by disturbance or fluctuating conditions (Lee and Gelembiuk 2008). For instance, in the St. Lawrence estuary, two genetically distinct clades of *E. affinis* (Fig. 2B, Atlantic, North Atlantic) overlap in geographic distribution, but the clade that gives rise to invasive populations predominates in microhabitats marked by seasonal fluctuations in salinity (Winkler et al. 2008).

Balancing selection in the form of fluctuating selection over time could help maintain genetic variation in the native saline range. With the presence of antagonistic pleiotropy between saline and freshwater tolerance, temporally varying selection acting across generations would select for different genotypes at different generations (Turelli and Barton 2004; Lee and Gelembiuk 2008; Posavi et al. 2014). This pattern of selection would greatly contribute to the maintenance of both saline‐ and freshwater‐adapted genotypes in the population.

The presence of overlapping generations expands the conditions under which fluctuating selection could protect polymorphism, by preserving genotypes that had been subjected to different selection regimes across generations (Hedrick 1995; Ellner and Sasaki 1996). In the case of *E. affinis*, a high degree of generational overlap occurs in the form of seasonal diapause eggs (Ban and Minoda 1992). Such diapausing eggs of *E. affinis* could survive in the sediment at least for several decades (Katajisto 1996) and become injected into the population at a later time.

Moreover, in temporally varying habitats, the parameter range under which polymorphism is preserved and the efficiency with which antagonistically selected alleles are maintained could be greatly expanded with the presence of ‘beneficial reversal of dominance’ (Curtsinger et al. 1994; Connallon and Clark 2012; Posavi et al. 2014). Beneficial reversal of dominance refers to the case where dominance switches at a locus across distinct traits (e.g. fitness in different environments), such that an allele is always dominant for the trait where it is beneficial and recessive where it is harmful. Beneficial reversal of dominance would result in freshwater tolerance being dominant under freshwater conditions and saltwater tolerance being dominant under saline conditions. Such a pattern of dominance would arise when the favoured allele in the heterozygote compensates for the lowered function of the less‐favoured allele in each environment, so that fitness of the heterozygote resembles that of the favoured homozygote (Wright 1929; Kacser and Burns 1981).

In such a situation, the less‐fit alleles would always be masked from selection in the heterozygous state, preventing erosion of genetic variation for salinity tolerance in both saline and freshwater environments (Wallace 1968; Hoekstra et al. 1985; Curtsinger et al. 1994). Such reversal of dominance would explain the maintenance of alleles favouring high‐salinity tolerance in a decades‐old freshwater population and of alleles favouring freshwater tolerance in a saline population, despite the presence of trade‐offs between fresh and higher salinity tolerance (Lee et al. 2003, 2007).

**Figure 4:**
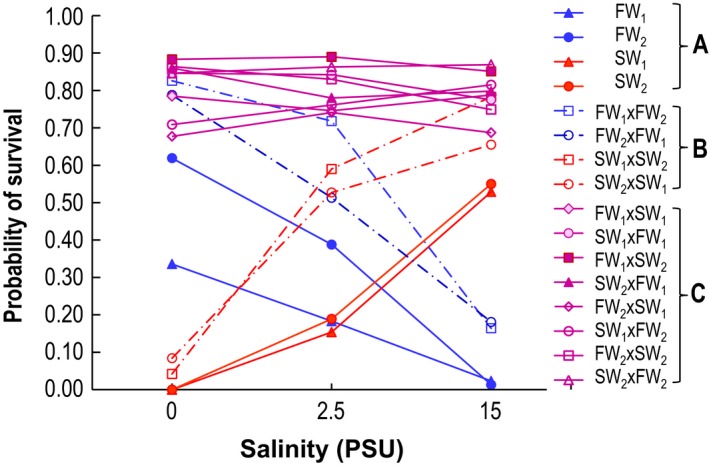
Demonstration of beneficial reversal of dominance in salinity tolerance across salinities in the copepod *Eurytemora affinis*. Maximum‐likelihood estimates of probabilities of survival from hatching to adult for (A) two freshwater and two saltwater parental inbred lines (matings within inbred lines), (B) reciprocal within‐salinity F1 crosses, between inbred lines independently derived from a given population (to account for heterosis), and (C) reciprocal between‐salinity F1 crosses. Survival of the between‐salinity F1 crosses (C, purple lines) was not significantly different from survival of freshwater F1 crosses (B, blue dashed lines) under freshwater conditions or survival of saltwater F1 crosses (B, red dashed lines) under saltwater conditions. This pattern of survival strongly supported the presence of beneficial reversal of dominance. Adapted from Posavi et al. (2014).

The first explicit and rigorous test for the presence of beneficial reversal of dominance demonstrated strong evidence for the switching of dominance in salinity tolerance across salinity conditions using inbred lines of *E. affinis* (Fig. 4) (Posavi et al. 2014). To test for beneficial reversal of dominance, crosses were performed between and within saline and freshwater inbred lines and their survival was compared across salinities. Multiple inbred lines were independently generated from both saline and freshwater populations, through full‐sibling mating for 30 generations. Crosses between saline and freshwater inbred lines would be heterozygous at loci that would confer saline or freshwater tolerance, such that comparing survival of these between‐salinity crosses to survival of the parental inbred lines (mating within inbred lines) would uncover the switching of dominance in salinity tolerance between saline and freshwater conditions.

Support for beneficial reversal of dominance was evident from the fact that survival of F1 crosses between the saltwater and the freshwater inbred lines (Fig. 4C, purple) was not different from that of saltwater F1 crosses (salt × salt; Fig. 4A, red) under saltwater conditions (15 PSU), and from that of freshwater F1 crosses (fresh × fresh; Fig. 4A, blue) under freshwater conditions (0 PSU) (Posavi et al. 2014). That is, the heterozygote (Fig. 4C) displayed survival similar to the more‐fit homozygote in each environment. Moreover, the switching of dominance evident in the saline x freshwater F1 crosses (Fig. 4C) could not be explained by heterosis, which was accounted for by using crosses between independently derived inbred lines from each population (Fig. 4B).

This study using *E. affinis* provides a rare empirical example of complete beneficial reversal of dominance associated with environmental change (Posavi et al. 2014). This mechanism might be crucial for maintaining genetic variation in salinity tolerance within *E. affinis* populations, allowing rapid adaptation to salinity changes during habitat invasions. Reversal of dominance would enhance the maintenance of genetic variation under fluctuating conditions within native ranges by continuously protecting the maladapted alleles in the recessive state against negative selection. Upon invasions into fresh water habitats, reversal of dominance should, by rendering any freshwater‐beneficial alleles dominant in fresh water, increase initial rates of survival for stocks of *E. affinis* transplanted from the native saline range. By now rendering the saltwater‐beneficial alleles recessive in fresh water, the possibility still exists for the freshwater populations to later return to saline habitats, provided too much canalization has not occurred.

## Physiological evolution: evolution of body fluid regulation and the evolution of ion transporter expression and activity

**Figure 5:**
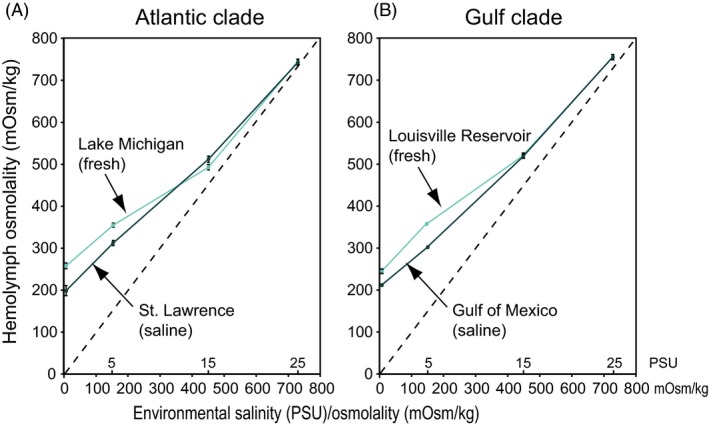
Evolutionary shifts in hemolymph osmolality from saline to freshwater habitats in the copepod *E. affinis*. Parallel evolutionary shifts were apparent for independent invasions in the two genetically distinct (A) Atlantic and (B) Gulf clades. The freshwater populations (light lines) from Lake Michigan and Louisville reservoir showed significantly elevated hemolymph osmolalities at lower salinities (0.2 and 5 PSU) relative to their saline ancestors (dark lines) from the St. Lawrence salt marsh and Gulf of Mexico (Tukey‐Kramer, *P* < 0.001). The dashed lines represent the isosmotic line, where the environmental and hemolymph osmolalities would be equivalent. The independent axis shows environmental osmolality in mOsm/kg and salinity in PSU (practical salinity units ≈ parts per thousand). The data points are mean osmolality ± standard error for 10–17 adult females. Adapted from Lee et al. (2012).

On macroevolutionary timescales, colonizing habitats removed from the sea required the evolution of increased physiological regulation to maintain body fluid homoeostasis against inhospitable environments (Willmer et al. 2004). Concordant with this trend, *E. affinis* populations display the evolution of increased body fluid regulation following freshwater invasions. This increased regulation is evident from the evolution of increased hemolymph osmolality at lower salinities in freshwater populations of *E. affinis*, relative to their saline ancestors (Fig. 5) (Lee et al. 2012).

When ancestral saline and freshwater invading populations of *E. affinis* were reared across a range of common‐garden salinities (~0–25 PSU), freshwater populations showed elevated hemolymph osmolality (by 16–31%) at lower salinities (below 15 PSU) (Fig. 5). Moreover, the same evolutionary increases in hemolymph osmolality occurred across two independent saline to freshwater invasions within genetically distinct clades of *E. affinis* (Lee et al. 2012) (Fig. 5).

Maintaining elevated body fluid concentrations with declining environmental salinity (Fig. 5, dark green lines) would impose serious challenges for a small organism such as *E. affinis* (<2 mm in length), where large surface area relative to volume could result in high rates of ion efflux. The fact that the freshwater populations maintain an even higher ionic gradient with the external freshwater environment than the saline populations (Fig. 5, light teal lines) indicates an even greater challenge. This shift towards greater body fluid concentrations at low salinity would entail increased energetic costs upon invading freshwater habitats (see section *Cost of adaptation? Energetic cost and the evolution of salinity tolerance* × *food requirement*) (Lee et al. 2013).

Maintaining elevated body fluid concentration (hemolymph osmolality) would require increases in ion uptake and/or reduction in ionic losses. In the copepod *E. affinis*, experimental results indicate that freshwater invasions are accompanied by the evolution of ion transport function, with increases in ion uptake under freshwater conditions (Lee et al. 2011). Ion transport is ubiquitous in all cells and across all domains of life and is essential for maintaining cell homoeostasis and a variety of functions, such as nutrient uptake, cell volume regulation and neuronal signal transduction. All cells contain primary ion transporters, such as Na^+^/K^+^‐ATPase (NKA) and V‐type H^+^ ATPase (VHA), which power the transport of ions across cell membranes using ATP.

**Figure 6:**
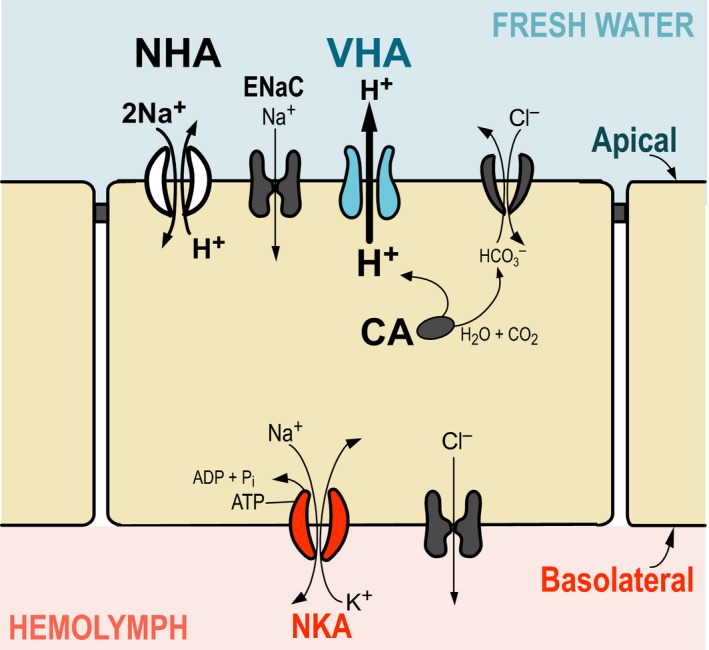
Model of ion uptake by an ionocyte in the copepod *E. affinis*, showing a set ion transporters that might be important for ion uptake from fresh water. In freshwater habitats, where ionic concentration of the water is extremely low, ions (such as Na^+^) must be transported into the cell against a steep concentration gradient. V‐type H^+^ ATPase (blue) is localized on the apical (outer) membrane of the cell (Gerber et al. 2015), to generate an electrochemical potential by pumping H^+^ out of the cell. This proton gradient then drives the transport of Na^+^ into the cell *via* secondary transporters, possibly electrogenic Na^+^/H^+^ antiporter (NHA) or Na^+^ channel (ENaC). Na^+^ is then pumped across the basolateral membrane to the hemolymph by Na^+^/K^+^‐ATPase (red).

The ion transporter V‐type H^+^‐ATPase (VHA) has been hypothesized to drive the uptake of cations against a steep concentration gradient in low‐salinity environments (Ehrenfeld and Klein 1997; Wieczorek et al. 1999; Beyenbach 2001; Weihrauch et al. 2001; Patrick et al. 2006; Tsai and Lin 2007). In models of strongly hyperosmoregulating crustaceans, this enzyme is localized on the apical membrane of epithelial cells, where it could create a proton gradient by pumping H^+^ ions out of the cell (Fig. 6). This transmembrane potential generated by VHA could then energize the uptake of Na^+^ *via* secondary transporters (such as the Na^+^ channel [ENaC] or an electrogenic Na^+^/H^+^ antiporter [NHA]). These ions are then transported to the hemolymph *via* NKA (Day et al. 2008; Charmantier et al. 2009; McNamara and Faria 2012; Xiang et al. 2012).

**Figure 7:**
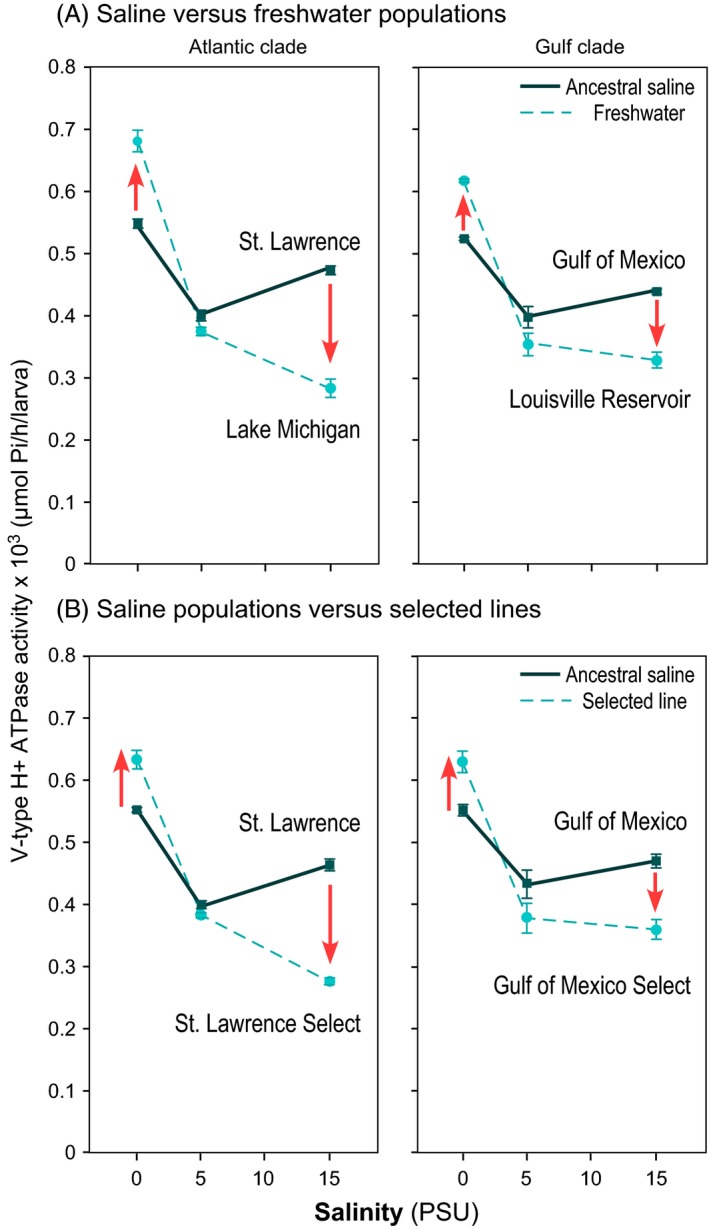
Evolutionary shifts in ion transport enzyme V‐type H^+^ ATPase activity between saline and freshwater populations from the Atlantic (left graphs) and Gulf clades (right graphs) of the copepod *E. affinis*. (A) Enzyme activity of ancestral saline populations relative to natural freshwater populations. (B) Enzyme activity of ancestral saline populations relative to freshwater‐selected populations, where saline populations were selected for freshwater tolerance over 12 generations in the laboratory. Arrows indicate evolutionary shifts in the derived freshwater populations (dashed lines), towards increased enzyme activity in fresh water (~0 PSU, Lake Michigan water) and reduced activity at high salinity (15 PSU) relative to the ancestral saline populations (solid lines). Data points are mean ± SE for six replicate treatments. Data were taken from Lee et al. (2011).

Concordant with this model, freshwater populations of *E. affinis* were found to exhibit evolutionary shifts in V‐type H^+^ ATPase (VHA) activity and expression relative to their saline ancestors (Fig. 7) (Lee et al. 2011). Because saline and freshwater populations were reared under a set of common salinities (0, 5, or 15 PSU) to remove effects of environmental acclimation (i.e. population differences due to developmental plasticity), differences in enzyme activity and expression found between the populations reflect genetically based (i.e. evolutionary) changes. In particular, freshwater populations showed increases in activity and expression of the ion transport enzyme VHA in fresh water (0 PSU, lake water) and declines at higher salinity (15 PSU), relative to the saline ancestral populations (Lee et al. 2011). This evolutionary shift occurred in parallel in two genetically distinct clades (Fig. 7A). This pattern following freshwater invasions, of increased VHA function in fresh water and declines at higher salinity, is consistent with the evolutionary trade‐offs found between survival at low *versus* high salinities (see section *Evolution of physiological tolerance and plasticity during invasions*; Fig. 3).

In addition, the same pattern of VHA evolution was found in laboratory selection experiments, where ancestral saline populations were subjected to selection for fresh water tolerance for ~12 generations (Fig. 7B) (Lee et al. 2011). The same evolutionary shifts in VHA activity was found for freshwater‐selected lines derived from genetically distinct saline populations, as well as for selected lines derived independently from the same ancestral saline population. Thus, parallel evolution was evident among laboratory selection lines derived from both within and between genetically distinct ancestral populations. Moreover, laboratory selection was able to recapitulate the evolutionary shifts found in the wild populations.

In contrast to the pattern found for VHA, the ion transport enzyme Na^+^/K^+^‐ATPase (NKA), previously considered to be the principal driving force for ion uptake from the environment (Lucu and Towle 2003; Kirschner 2004), showed declines in activity and expression across all salinities in the freshwater populations, relative to their saline ancestors (Lee et al. 2011). As in the case for VHA, these evolutionary shifts were repeatable, with parallel evolutionary shifts across independent invasions in the wild, as well as in selection experiments in the laboratory (Lee et al. 2011).

Results on ion transport evolution in *E. affinis* are important as the first demonstration of VHA evolution in response to salinity change, supporting prevailing hypotheses regarding ion uptake from dilute environments (Charmantier et al. 2009; Lee et al. 2011; McNamara and Faria 2012). Moreover, the speed of these evolutionary shifts is remarkable, of only a few generations (~12) in the laboratory and a few decades (~60 years) in the wild. The parallel evolutionary shifts across multiple independent invasions from genetically distinct clades in the wild, and in laboratory selection lines, reveal labile evolutionary mechanisms underlying these rapid evolutionary events. The evolutionary parallelism observed here could potentially have general relevance for many other aquatic invaders from brackishwater habitats (e.g. zebra and quagga mussels and other Ponto‐Caspian invaders) (Jażdżewski 1980; Taylor and Harris 1986; Dietz et al. 1996; Lee and Bell 1999; Ricciardi and MacIsaac 2000).

## ‘It's all in the legs’: localization of ion transporter expression in the legs

**Figure 8:**
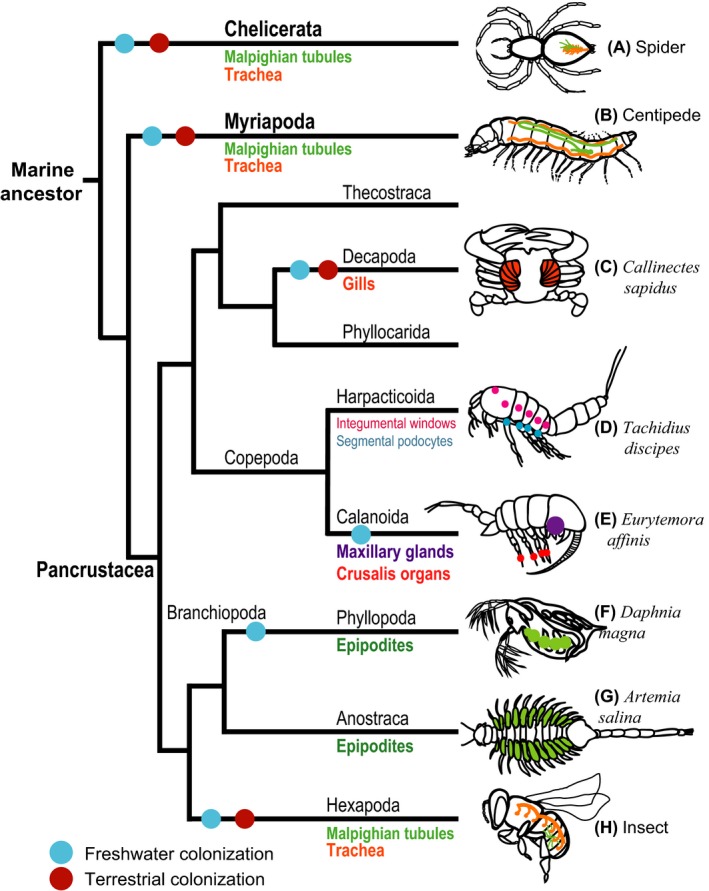
Evolutionary history of osmoregulatory structures and habitat colonizations in the phylum Arthropoda. Morphological and genetic data suggest that at least some of the osmoregulatory and respiratory structures of aquatic and terrestrial arthropods are derived from appendages and are homologous, such as the epipodites, gills, and trachea within the Pancrustacea. Homology among the osmoregulatory and respiratory organs of the subphyla, Chelicerata (e.g. spiders, A), Myriapoda (e.g. centipedes, B), and Pancrustacea (specifically Hexapoda, H), is not known. Most of the terrestrial colonizations are thought to have occurred *via* freshwater intermediate habitats. Phylogenetic topology is based on Meusemann et al. (2010), von Reumont et al. (2012), and Oakley et al. (2013).

The arthropods have been particularly successful in exploiting diverse habitats, and radiating into the most abundant and speciose phylum. Three subphyla (Pancrustacea, Myriapoda and Chelicerata) have independently colonized freshwater habitats and land (Fig. 8) (Meusemann et al. 2010; Regier et al. 2010; von Reumont et al. 2012). The most well studied subphylum is the Pancrustacea (i.e. insects, crustaceans), where marine ancestors colonized freshwater habitats multiple times independently. Within the Pancrustacea, the hexapods (i.e. insects) emerged from their crustacean (likely Branchiopod‐like) ancestors to display their extraordinary success in colonizing diverse terrestrial habitats and radiating into an incredible diversity of forms (Glenner et al. 2006; Meusemann et al. 2010; von Reumont et al. 2012; Oakley et al. 2013).

Based on studies within the Pancrustacea, for which the most information is available, these colonizations into freshwater and terrestrial habitats were facilitated by innovations that arose from the evolution of development of appendages (e.g. leg organs). That is, based on developmental and morphological studies, arthropod appendages are posited to have evolved into homologous osmoregulatory and respiratory structures in marine, freshwater and terrestrial habitats (Fig. 8) (Hrycaj and Popadic 2005; Franch‐Marro et al. 2006; Boxshall and Jaume 2009). While arthropod appendages could take on the form of legs, antennae, maxillae and wings, they are also thought to be homologous with osmoregulatory and respiratory structures, such as epipodites of ‘lower crustaceans’ (e.g. *Daphnia*), gills of decapods (e.g. crabs), and tracheal systems of terrestrial arthropods (Hrycaj and Popadic 2005; Franch‐Marro et al. 2006; Boxshall and Jaume 2009).

**Figure 9:**
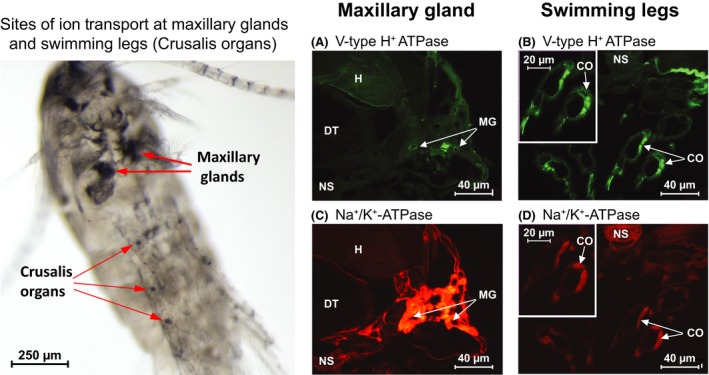
Sites of ion transport in the copepod *Eurytemora affinis*. Left: Silver Staining of the whole animal, showing sites of ion exchange at the maxillary glands and swimming legs (in the Crusalis organs). Right: Immunohistochemical localization of ion transport enzyme expression in the maxillary gland (A, C) and swimming legs (B, D). V‐type H^+^ ATPase (VHA) expression: (A) Maxillary Gland, showing green fluorescent staining for VHA on the apical membranes and (B) Swimming Legs, where VHA staining was strong. Na^+^,K^+^‐ATPase (NKA) expression: (C) Maxillary Gland, showing very strong red fluorescent staining for NKA on the basolateral membranes and (D) Swimming Legs, where NKA staining was relatively weak. DT = digestive tract, H = hepatopancreas, MG = maxillary glands, NS = nervous system, CO = Crusalis organs. Adapted from Johnson et al. (2014).

**Figure 10:**
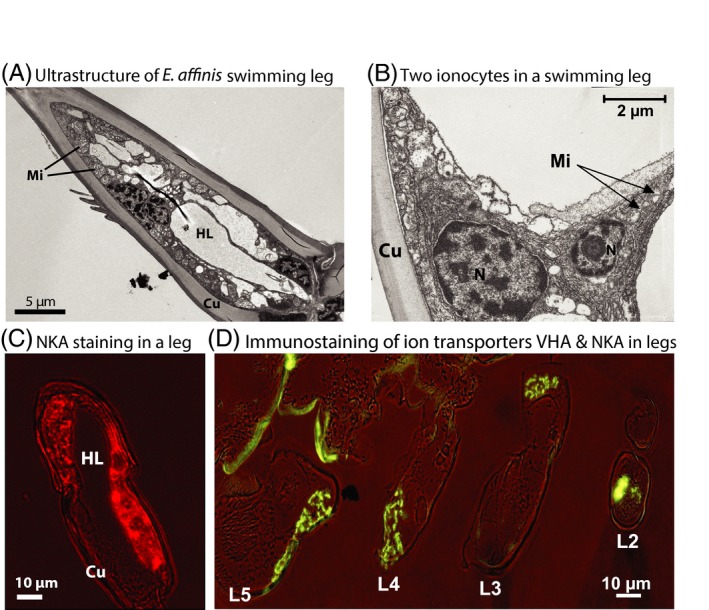
Ultrastructure of the ion regulatory Crusalis organs in the swimming legs of the copepod *E. affinis*. (A) Transmission electron micrograph of a swimming leg article, showing internal structure. (B) Ionocytes, cells specialized for ion transport, within the Crusalis organ. (C) Immunostaining of Na^+^,K^+^‐ATPase (red) in a swimming leg, showing the Crusalis organ occupying much of the volume of the leg article. (D) Immunostaining of both ion transporters V‐type H^+^ ATPase and Na^+^,K^+^‐ATPase (green) in the Crusalis organs of four swimming legs of a copepod (L2–L5). HL: Hemolymph Lacuna, Mi: Mitochondria, N: Nucleus, Cu: Cuticle. Adapted from Johnson et al. (2014) and Gerber et al. (2015).

Concordant with cases of physiological innovation deriving from arthropod appendages, the sites of ion uptake in the copepod *Eurytemora affinis* were found to occur in their swimming legs (Figs 9 and 10) (Johnson et al. 2014; Gerber et al. 2015). These novel ion regulatory leg organs were described by the laboratories of Carol Eunmi Lee and Guy Charmantier and named the ‘Crusalis organs’ (Johnson et al. 2014). Based on quantification of *in situ* expression of ion transporters and histological observations, the sites of ionic and osmotic regulation were localized to the maxillary glands and the Crusalis organs (Figs 9 and 10).

A variety of results implicate these Crusalis swimming leg organs as the main sites of ion uptake in freshwater habitats (Johnson et al. 2014; Gerber et al. 2015). *In situ* immunohistological expression of the ion transporter V‐type H^+^ ATPase (VHA) was much more intense in the Crusalis leg organs relative to the maxillary glands (Fig. 9A versus B). Additionally, VHA expression in the Crusalis organs was significantly higher in the freshwater population (at 0 PSU) relative to the saline population (at 15 PSU) (Gerber et al. 2015), consistent with the importance of VHA function in fresh water. Increases in VHA expression in the swimming legs arose from an increase in the abundance of VHA per cell, rather than an increase in number of ionocytes, indicating a simple mechanism for increasing VHA function at low salinity (Gerber et al. 2015).

In contrast, *in situ* immunoexpression of Na^+^/K^+^‐ATPase (NKA) was extremely high in the maxillary glands, consistent with the hypothesized excretory function of these glands (Fig. 9C) (Johnson et al. 2014). In addition, NKA expression was also elevated in the Crusalis organs in the saline population under saline conditions, indicating that the Crusalis leg organs are likely important for ion transport in saline, as well as freshwater, habitats (Gerber et al. 2015). The decline in NKA expression in the freshwater population arose from decreases in number or size of ionocytes containing NKA, rather than from changes in number of NKA per cell, suggesting resource conservation with changes in salinity (Gerber et al. 2015).

The aggregate of results above paints a consistent picture of how ion transport function evolves at the protein, cellular and organ levels across salinities. These studies present a rare comprehensive analysis of the evolution of ion transport function. The proton pump VHA is clearly associated with ion uptake in low‐salinity habitats and is localized most heavily in the Crusalis leg organs. Ionocytes within the Crusalis organs express greater abundance of VHA under freshwater conditions, particularly on the apical membranes (Gerber et al. 2015). This increase in VHA expression in the Crusalis organs is likely a target of natural selection during freshwater invasions. Such results provide insights into mechanisms of ionic regulation for this species, with added insights into evolutionary mechanisms underlying physiological adaptation during habitat invasions.

## Cost of adaptation? Life history evolution

**Figure 11:**
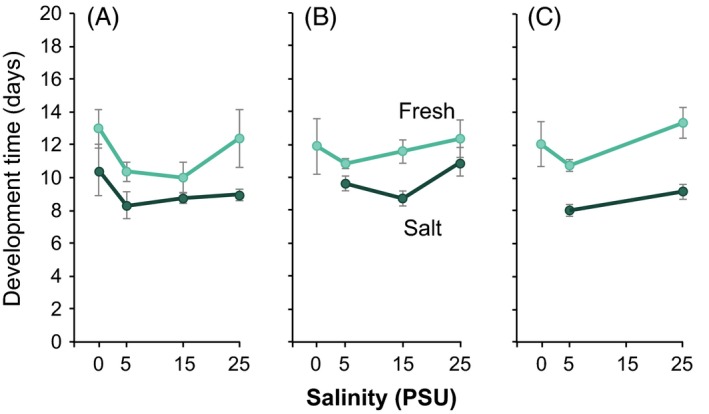
Evolutionary shifts in larval development time from the saline St. Lawrence (dark green) to freshwater Lake Michigan (teal) *E. affinis* populations, measured across salinities (0, 5, 15, and 25 PSU). Graphs show mean development time from hatching to metamorphosis (in days) ± SE for clutches taken from parental saline and freshwater populations that were reared at (A) native salinities, 15 PSU for saline and 0 PSU for freshwater populations (*n* = 8 clutches per population), for at least two generations prior to the reaction norm experiment (at 0, 5, 15, and 25 PSU), (B) 5 PSU salinity for two generations (*n* = 12 clutches), and (C) 5 PSU salinity for six generations (*n* = 14 clutches) prior to the reaction norm experiment. Results show that evolutionary differences in development time between saline and freshwater populations are robust to rearing salinities of the parental populations prior to the reaction norm experiments. Adapted from Lee et al. (2007).

Along with the evolution of physiological tolerance and performance (previous sections), we also observed the evolution of life history traits in recently derived freshwater populations of *E. affinis* (Lee et al. 2003, 2007). Relative to their saline ancestors, the freshwater populations showed retarded development before metamorphosis, during the larval (naupliar) life history stage (Fig. 11), but not after metamorphosis (Lee et al. 2003, 2007). The evolutionary shift in development time was quite robust to the rearing conditions of the parental saline and freshwater populations (Fig. 11). Three independent experiments, where the parental populations were reared at the intermediate compromise salinity of 5 PSU for differing numbers of generations (0, 2, 6) prior to the common‐garden reaction norm experiments, yielded the same pattern of evolution of development time (Fig. 11).

These evolutionary shifts might arise as trade‐offs between freshwater tolerance and development rate. The retarded development before metamorphosis observed in the freshwater population might result from a need to build the physiological machinery for increased tolerance and performance under freshwater conditions. Retarded development at the larval stages is consistent with most mortality due to osmotic stress occurring at larval stages (Lee et al. 2003). This retarded development associated with freshwater adaptation in *E. affinis* follows the same trend as the extended development times found in ancient freshwater‐adapted copepod species (Peterson 2001). Also, the longer development times are concordant with research showing a positive correlation between selection for stress resistance and increase in longevity and development time in *Drosophila melanogaster* (Rose et al. 1992; Archer et al. 2003).

## Cost of adaptation? Energetic cost and the evolution of salinity tolerance × food requirement

Given the serious physiological challenges imposed by invading freshwater habitats, why are many brackishwater species so successful as invaders in freshwater environments? The energetic demands of freshwater adaptation must be high, given the increased machinery required for ion transport, such as increased activity and expression of V‐type H^+^ ATPase, which consumes ATP (see previous sections). Such increases in the need for ion uptake in freshwater habitats would likely lead to increases in food requirements to fuel this energetic cost.

Given their energetic requirements, brackishwater species might be constrained from invading freshwater habitats due to lack of adequate amount of particular types of food (Lee et al. 2013). Eutrophic conditions have been proposed to promote invasions into freshwater habitats, due to the enormous productivity of invasive species and their competitive advantage under high resource conditions (Engelhardt 2011). Freshwater invaders of brackish origins, such as the zebra mussel *Dreissena polymorpha* and the amphipod *Corophium curvispinum*, have the added burden of having less efficient ionoregulatory capacities and the need for high rates of ion uptake under freshwater conditions (Taylor and Harris 1986; Horohov et al. 1992; Dietz et al. 1996; Lee et al. 2011, 2012). Such freshwater invaders of brackishwater ancestry, such as zebra mussels and the copepod *Eurytemora affinis*, have been found to have voracious appetites when residing in freshwater habitats (MacIsaac et al. 1992; Tuchman et al. 2004; Lee et al. 2013). In addition, these invaders possess strong preference for algae that are high in particular long‐chain polyunsaturated fatty acids, such as cryptophytes, when in residing in fresh water (Vanderploeg et al. 1996).

**Figure 12:**
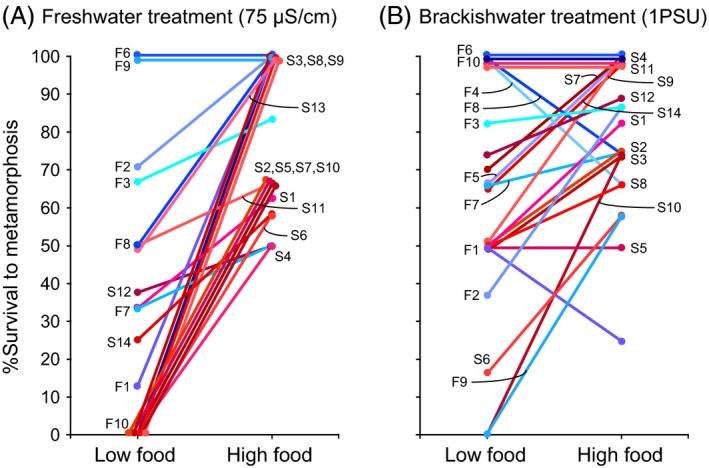
Impacts of food concentration and salinity on survival of clutches (genotypes) in the saline (red, St. Lawrence) and freshwater (blue, Lake Michigan) populations of the copepod *E. affinis*. **%**Survival from hatching to metamorphosis is shown for individual clutches reared at (A) low salinity (fresh water, 75 *μ*S/cm) and (B) higher salinity (brackish water, 1 PSU), in response to low food (700 cells/mL) and high‐food concentrations (14 000 cells/mL). Individual reaction norms are shown for 8–10 clutches from the freshwater population (blue, F1–F10) and 13–14 clutches from the saline population (red, S1–S14). Higher food concentration had a much greater impact on enhancing the survival of the saline population (red lines), particularly at the low‐salinity condition (A, left graph). Adapted from Lee et al. (2013).

So does higher concentration of food, of the correct type, enhance the ability of brackishwater populations to expand their ranges into freshwater habitats? Common‐garden experiments on saline and freshwater populations of the copepod *Eurytemora affinis* revealed that high‐food concentration (of cryptophytes) was able to significantly increase low‐salinity tolerance in both populations (Fig. 12A) (Lee et al. 2013). This rescue effect of high‐food concentration was far more pronounced under freshwater conditions (Fig. 12A) than at a higher salinity (Fig. 12B).

Interestingly, the beneficial impact of high‐food concentration was greatly reduced in the freshwater population (Fig. 12, blue lines), relative to its saline ancestor (red lines) (Lee et al. 2013). This result indicates that the freshwater population evolved the ability to become far less dependent on high‐food concentrations in order to survive freshwater conditions. Conversely, the saline population showed very high dependency on high‐food concentration for surviving under freshwater conditions (Fig. 12A). Thus, an excess of food could enhance the ability of the saline population to survive freshwater conditions and extend its range into freshwater habitats, allowing subsequent evolution of low‐salinity tolerance even under food‐poor conditions.

In addition, impacts of food concentration on freshwater survival differed significantly between different brackish populations from the native range, suggesting that insufficient food concentration could pose a key barrier to range expansions for certain populations more than others (Lee et al. 2013). Two brackish populations occur in close proximity in the St. Lawrence estuary, one from an invasive clade (Fig. 2, Atlantic clade, red) and another from a noninvasive clade (Fig. 2, North Atlantic clade, teal). These clades differ in their microhabitat distributions such that the invasive clade tends to be exposed to greater environmental fluctuations than the noninvasive clade (Winkler et al. 2008). Freshwater survival at low food concentration was significantly greater in the population from the invasive clade than from the noninvasive clade. Thus, while the ancestral saline population requires significantly higher food concentration to tolerate low salinities than the freshwater Lake Michigan population (Fig. 12), the saline population from the noninvasive clade requires even more.

This study is the first to examine the role of food concentration, as well as the interaction between food concentration and salinity tolerance, in facilitating saline to freshwater invasions. Salinity is a major constraint in limiting invasions by brackish species into freshwater habitats, but high‐food concentration (of the correct type) reduces the constraint, such that food x salinity interaction (the impact of food concentration on salinity) greatly extends range limits into freshwater habitats. Thus, high‐food concentration (of the correct type) could serve as an equalizer, in allowing species of saline ancestry, such as zebra mussels, to colonize novel freshwater environments and compete with resident freshwater species. With sufficient food, saline species tend to grow faster and have higher fecundity relative to comparable freshwater species (Anger 1995; Peterson 2001). This phenomenon might be viewed as a type of ‘condition‐specific competition’ (Dunson and Travis 1991; Taniguchi and Nakano 2000), where gradients in food and salinity could allow brackish invaders and freshwater residents to coexist in the freshwater environment.

## Conclusions: insights gained from the study of the copepod *Eurytemora affinis*

Studies on the copepod *Eurytemora affinis* demonstrate that detailed evolutionary and physiological studies could offer profound insights into mechanisms of invasive success. For instance, comprehensive phylogeographic analysis of *E. affinis* populations revealed the incidence of cryptic invasions, as well as independent invasions crossing habitat boundaries (Lee 1999, 2000; Lee and Frost 2002). Phylogeographic and population genetic studies also revealed a considerable amount of heterogeneity in the native range, as well as the presence of noninvasive populations and clades (Lee 1999; Winkler et al. 2008). The ability to rear *E. affinis* populations over multiple generations in the laboratory enabled the demonstration of rapid evolution of physiological traits in response to environmental change (Lee et al. 2003, 2007, 2011, 2012, 2013; Posavi et al. 2014). These experiments revealed parallel evolution that was both repeatable in the laboratory, as well as in wild populations. The use of genetically inbred lines made it possible to uncover a mechanism that could contribute greatly to the maintenance of genetic variation within populations, with important implications for the evolutionary potential of invasive populations (Posavi et al. 2014). Detailed *in situ* gene expression studies identified the specific localization of traits undergoing evolutionary change during habitat invasions (Johnson et al. 2014; Gerber et al. 2015). Many of the studies described in this review represented among the earliest of their kind, and the aggregate of these studies contributed to the growing literature on evolutionary mechanisms of habitat invasions (reviewed in Lee 2002; Lee and Gelembiuk 2008; Lee 2010). Moreover, insights gained from these studies are furthering our understanding of the evolutionary potential of populations and factors leading to their invasive success (Lee and Gelembiuk 2008; Lee 2010; Hufbauer et al. 2012).

In addition to the studies above, the Lee Lab is currently identifying evolutionary changes in gene expression during saline to freshwater invasions that resulted from *cis*‐regulatory changes, due to mutations near the gene (e.g. promoter or enhancer driving expression), as opposed to *trans*‐regulatory changes, due to mutations somewhere else in the genome (e.g. transcription factor) (Posavi 2015). Loci that show evolutionary increases in expression due to *cis*‐regulatory changes during freshwater invasions are likely to have been targets of natural selection during freshwater invasions, indicative of a critical role in freshwater adaptation (Wittkopp et al. 2004; Wray 2007).

In another study, the Lee Lab has found that the copepod *E. affinis* is inhabited by an enormous consortium of microbiota. Interestingly, there is a dramatic shift in the copepod microbiome during invasions, with parallel shifts in the microbiome composition during saline to freshwater invasions (Gelembiuk et al. 2015). For instance, the family Rhodobacteraceae declines in abundance during parallel freshwater invasions by the copepod host, whereas the *Rubrivivax* subgroup within the family Comamonadaceae increases dramatically during these invasions. The copepod microbiome could potentially play important roles in freshwater adaptation during invasions. This is a topic that warrants further investigation.

Considerable attention has been devoted to identifying characteristics of successful invaders, such as life history, phenological, and reproductive traits (Ehrlich 1986, 1989; Williamson and Fitter 1996; Kolar and Lodge 2001). However, the ability to predict invasions could be enhanced if efforts to characterize successful invaders incorporated information pertaining to the evolutionary properties of invasive populations. For instance, if it were known that a particular trait consistently evolves during invasions in particular invaders, quantitative genetic variation for those critical traits would be highly informative. As invasive species are often composed of highly heterogeneous populations or sibling species, phylogeographic information would be useful for tracking the specific invasive populations that actually pose a threat. Given that the evolutionary potential of populations is influenced by the selection regime and evolutionary history in the native range (Lee and Gelembiuk 2008), characteristics of the native ranges (e.g. disturbance, environmental fluctuations, spatial heterogeneity) could provide useful information on the potential geographic sources that are likely to give rise to invasive populations. In short, examining invasive species through an evolutionary lens could, in conjunction with ecological information, provide powerful insights regarding populations that are likely to invade.

## Data archiving statement

As this paper is a review, there are no data to archive.

Box 1 **Personal Reflections**

For this invited submission, I have been asked to provide my personal reflections on being a woman in science. I had the opportunity to reflect on my life and career a few years back, while diving in a deep lake in Turkey. At that time, I became ensnared in a Japanese fishing net abandoned at the bottom of Lake Bafa, at the small town of Herakleia. The net was riddled with tenacious barbs that clung onto my wetsuit, clinging more tightly the more I moved. At the bottom of the cold and dark lake, I had time to think for 20 min, which felt like a really long time. I felt immense gratitude for the privileges I have had, and how I have been able to fulfil most of my dreams and aspirations. And, moreover, I was incredibly grateful for the extremely sharp dive knife gifted to me by the SCUBA equipment company Wenoka, without which I would not be here to share my reflections (thank you Wenoka!).

Reflecting on my past, I would have to say that nothing I encountered in my professional life could match the challenges I confronted as a child. I was essentially immunized from any insults later on in life by developing awareness of my identity during my early years. I was born in Champaign, Illinois, where my father worked on his Ph.D. in physics. My deep interest in science originated from that time, as my first memory in life was the *Tyrannosaurus rex* posing quite grandly in the foyer of the Field Museum in Chicago. From that point on, I was enamoured by geology, natural history and evolution.

From there my family moved to Montreal, where we lived for ~7 years. In terms of exploring my interests, this period was the most difficult. The greatest barrier to pursuing my interests was not the society or culture at large, but my mother's personal desire to recreate her image in me. As a consequence, most of my childhood was spent on activities that I found arduous or tedious, such as demanding ballet lessons that I did not want. She discouraged my interest in science, giving away my rock collection and dinosaur books. It would have been nice to have my mother's will buffered by my kinder physicist father, but he was extremely busy and consumed by his work. Although my brother resembled my mother more closely in personality and interests, my mother was extremely laissez‐faire with him and did not regard him as an appropriate reflection of herself, given that he was male. While my mother was extreme, I think the tendency to stereotype and impact the fate of someone based on sex is not an uncommon practice. All too often I have observed people ascribing properties and characteristics to people based on their sex, rather than on their actual aptitudes or personalities.

An important watershed moment arrived when my father obtained a faculty position in Seoul, Korea when I was nine years old. This move was critical in expanding my world beyond the confines of my mother's sphere, as I gained exposure to my extended family. I found important role models in my Aunts, my mother's elder sisters, particularly in Professor Yun Chung‐ok (윤정옥). She is well known for tracking down and drawing attention to the former sex slaves of the Japanese military that were abandoned across South East Asia after World War II. I had the benefit of spending time with her and listening to her accounts of history and literature. Her academic work focused on 19^th^ century British novels, with particular interest in Charles Dickens’ illustrations of inequality and poverty. Another influential role model was my paternal great grand uncle, Professor Lee Chongwoo (이종우). He was a gentleman born in the Joseon dynasty, and in the 1920s completed his graduate studies in Paris in the fine arts. I admired his sophistication and old world air, which contrasted sharply with Korea's fast paced frenzied high‐tech society. I had the chance to spend time in his art studio, watching him patiently and steadfastly paint, draw and write calligraphy.

As I showed no talent in dance, my mother switched gears and placed me in fine art classes to train for the entrance exams into a competitive arts middle/high school. At this arts school, I found my classmates to be bright, entertaining, wildly creative and at times astonishingly gifted. While my classmates could at times be volatile, I was happy to be inspired and entertained by them. During the many breaks between classes, I was able to wander between the music, art and dance departments and play with my friends on instruments and watch my classmates perform. It was fun sitting next to movie stars and then watch them on television or in film. To this day, I enjoy reading about my classmates in the *New York Times* Arts Section and watching them in films and TV dramas.

Despite the rich experiences, I was still a misplaced scientist who happened to be in an arts school. At this time, my mother was on television, hosting a show with a popular pop star. My family moved out of faculty housing into the flashy Cheongdam‐dong neighbourhood, in the Gangnam district of Seoul. This place has since become an entertainment and high‐tech centre, home to entertainment agencies, high‐tech software companies and world class EDM clubs. But when we lived here, this neighbourhood was mostly filled with high‐end boutiques with prohibitive prices. Here, I was subjected to more dance classes, this time with chic housewives wearing multicarat diamonds. The experiences during this time period were somewhat amusing, but often alienating, and typically ‘not me’.

At this time, the chance to pursue science seemed remote. Then, my father decided to go on sabbatical at Harvard, and my family relocated temporarily to the small provincial neighbourhood of Belmont, Massachusetts. Moving to the USA at age 17, I encountered for the first time racist stereotypes regarding Asians and Asian Americans. Not having grown up with these stereotypes, I had the immense benefit of not having internalized the false myths and media generated images. On the other hand, I was unprepared to deal with them and did not understand the origins of the stereotypes. I came to realize that the roles for Asian Americans in the USA were extremely canalized, professionally and socially, much more so than what I had experienced in Canada or Asia. Apparently, Asians and Asian Americans were viewed as subservient and robotic, appropriate for sidekick support staff roles, rather than as protagonists.

Stereotypes associated with being from Korea posed additional problems, given the depths of ignorance regarding the country. Ostensibly, I arrived from some tropical primitive country, where people run around barefoot and live in huts. As such, I was initially placed in lower level classes at Belmont High, rather than honours or Advanced Placement courses. At the same time, a classmate that arrived from Japan was placed into the advanced classes, despite having a weaker academic background than mine. I learned that perceptions of one's country of origin impacted how one is treated, and the degree of condescension that one encounters.

What I found even more appalling were the stereotypes specific to Asian women. I began hearing perplexing adjectives ascribed to me that I had never heard previously. For instance, people began telling me that, ‘you are really feminine’ without knowing me well. Was it my interest in mechanical toys or electronic gadgets, or the fact that I tended to dress in black? But then, when people got to know me better they would often say, ‘you are not the typical submissive Asian woman’, which was equally offensive. What on earth is such a woman? Like the assumptions made by my mother based on my sex, these comments reflected assumptions stemming from cultural baggage created by the American media and perhaps America's wartime history in Asia. Fortunately, I have found that the younger generations of Americans, particularly those born after the 1980s, have become less ignorant about Asia and less beholden to American stereotypes of Asia.

Towards the end of high school, I decided that I wanted to move to California, and set my eyes on attending Berkeley or Stanford. My mother then stepped in to try to take control over the process. She asserted that Berkeley and Stanford were not affordable; therefore, I had to attend a private women's college, one that was weaker in the sciences. This reasoning was in spite of the fact that the private women's college tuition was three‐fold that of Berkeley. So, I decided to opt for independence, and legally declared myself a financial independent. This legal status allowed me to report only my own income in college financial aid applications, and made Stanford quite affordable on my own. Consequently, I was able to make a clean break and become the full agent of my own life.

I became increasingly happy over time as I left my childhood behind me. My experience at Stanford was liberating and incredibly rewarding. I was able to experiment and find the things that I actually enjoyed doing. I met some fantastic people who listened to me and saw me for who I was, and made wonderful friends who were supportive and compassionate. In graduate school when my boyfriend obtained a faculty position and tried to convince me to take a subservient soft money position in his department, my housemate astutely asked, ‘does he even know who you are, after all these years?’ Apparently, he did not.

The sad reality is that sometimes the people closest to you are the very ones that try to inhibit your growth or progress. Despite close contact, even they could be blinded by preconceived notions and expectations of who you are and what you should become. I have been fortunate to transcend those limitations and escape from being boxed in. In turn, I try to put my best effort into seeing people for who they are, and try to question my own assumptions and biases. I think we would all benefit from being more thoughtful, more perceptive and more mindful, and able to look beyond the surfaces.
